# Microbial oil production from acidified glycerol pretreated sugarcane bagasse by *Mortierella isabellina*

**DOI:** 10.1039/c8ra08971j

**Published:** 2019-01-18

**Authors:** Guiqin Cai, Lalehvash Moghaddam, Ian M. O'Hara, Zhanying Zhang

**Affiliations:** Centre for Tropical Crops and Biocommodities, Queensland University of Technology GPO Box 2432, 2 George St Brisbane QLD 4001 Australia jan.zhang@qut.edu.au +61 7 3138 4132 +61 7 3138 7792

## Abstract

An integrated microbial oil production process consisting of acidified glycerol pretreatment of sugarcane bagasse, enzymatic hydrolysis, microbial oil production by *Mortierella isabellina* NRRL 1757 and oil recovery by hydrothermal liquefaction (HTL) of fungal biomass in fermentation broth was assessed in this study. Following pretreatment, the effect of residual pretreatment hydrolysate (containing glycerol) on enzymatic hydrolysis was firstly studied. The residual pretreatment hydrolysate (corresponding to 2.0–7.5% glycerol) improved glucan enzymatic digestibilities by 10–11% compared to the enzymatic hydrolysis in water (no buffer). Although residual pretreatment hydrolysate at 2.0–5.0% glycerol slightly inhibited the consumption of glucose in enzymatic hydrolysate by *M. isabellina* NRRL 1757, it did not affect microbial oil production due to the consumption of similar amounts of total carbon sources including glycerol. When the cultivation was scaled-up to a 1 L bioreactor, glucose was consumed more rapidly but glycerol assimilation was inhibited. Finally, HTL of fungal biomass in fermentation broth without any catalyst at 340 °C for 60 min efficiently recovered microbial oils from fungal biomass and achieved a bio-oil yield of 78.7% with fatty acids being the dominant oil components (∼89%). HTL also led to the hydrogenation of less saturated fatty acids (C18:2 and C18:3) to more saturated forms (C18:0 and C18:1).

## Abstract 2

A microbial oil production process consisting of acidified glycerol pretreatment of sugarcane bagasse, enzymatic hydrolysis, microbial oil production by *M. isabellina* NRRL 1757 and oil recovery by hydrothermal liquefaction of fungal biomass in fermentation broth was assessed.

## Graphical abstract

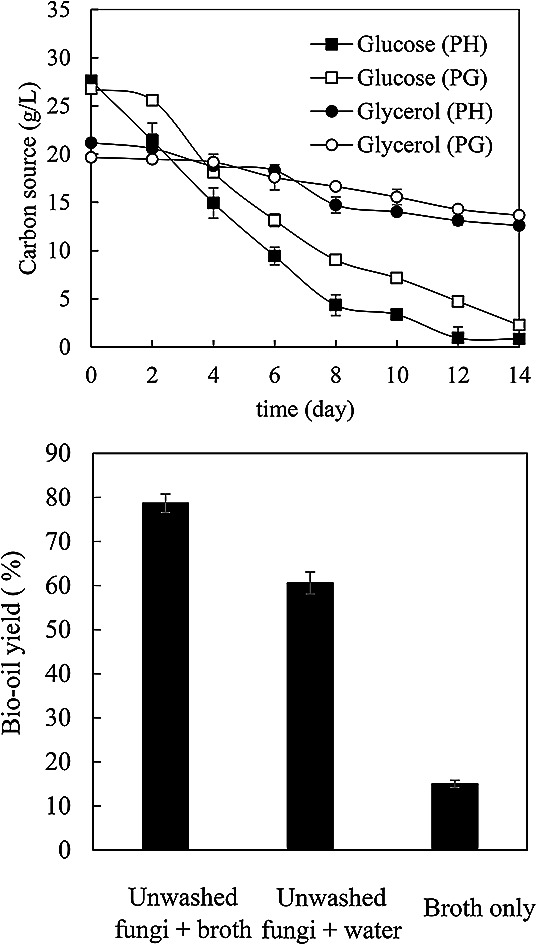

## 1. Introduction

Microbial oils produced by yeast and filamentous fungi are an alternative oil source to algal oils, vegetable oils and animal fat/oils for the production of biodiesels and advanced fuels such as jet fuels.^1,2^ In order to improve the process economics of microbial oil production, the use of low cost substrates such as glycerol and lignocellulosic biomass, and improvement of oil production (yield, concentration and productivity) has been recommended.^1,3^ Currently, glycerol is produced in large quantities with a low commercial value from the biodiesel industry (1 mole of glycerol is synthesized for every 3 moles of methyl esters produced) and the estimated yield is approximately 3.5 billion litres in 2018.^4,5^ Lignocellulosic biomass such as sugarcane bagasse is an abundant low cost carbon source. However, due to the recalcitrant nature of biomass fibres, pretreatment is required to deconstruct the biomass to improve sugars yield in a subsequent enzymatic hydrolysis step.^6^ Consequently, the pretreatment cost, the effect of biomass degradation products and co-conversion of mixed carbon sources need to be factored for microbial oil production by oleaginous microorganisms.^1,3^ In addition, as microbial oils are intracellular products, efficient and cost-effective processes are required to recover microbial oils for fuel production.

Numerous pretreatment methods including dilute acid, alkali, ionic liquids, organosolv and others have been reported to improve sugars yield from lignocellulosic biomass.^6,7^ Among these pretreatments, acid-catalysed glycerol-based organosolv pretreatment is one of the most effective pretreatment methods.^7,8^ Compared to dilute acid pretreatment, it is more effective in improving glucan digestibility.^8^ In addition, glycerol is cheap, biodegradable and environmentally friendly compared to conventional ionic liquids such as 1-butyl-3-methylimidazolium chloride.^8^ Glycerol-based pretreatment can be operated at atmospheric pressure because of its high boiling point (∼290 °C),^9^ thus leading to reduced capital cost on pressure resistant reaction equipment compared to pretreatment with low boiling point solvents such as ethanol.

For pretreatment with any high boiling point/non-volatile solvents (glycerol, ionic liquids, *etc.*), solvent recovery and recycling is critical to reduce the overall pretreatment cost. Washing of pretreated biomass for solvent recovery is necessary to reduce the solvent loss while incomplete biomass wash is preferred to avoid over-dilution of the solvent and reduce water usage. However, residual solvents may have negative effects on enzymatic hydrolysis and subsequent fermentation. The inhibitory effects of solvents were observed with ionic liquids and pure glycerol.^10,11^ Fortunately, glycerol was not detrimental to cellulase enzymes and it (up to 10%) did not inhibit ethanol production by yeast.^10^

On the other hand, as a low cost carbon source glycerol has been studied for producing succinic acids, 1,3-propanediol, ethanol as well as microbial oils by various microorganisms.^4,12,13^ Regarding microbial oil production, various yeast and filamentous fungi, such as *Rhodotorula*, *Rhodosporia*, *Yarrowia*, *Cryptococcus*, *Mucor* and *Mortierella*, were able to grow on glycerol.^12,14,15^ Furthermore, glycerol was also mixed sugars-derived from lignocellulosic biomass for the production of microbial oils by yeast strains, leading to improved microbial oil production due to improved C/N ratios.^16–19^ The studies with yeast strains such as *Yarrowia* and *Rhodosporidium* observed different metabolic mechanisms on consumption of glycerol–sugars mixtures. For example, *Rhodosporidium toruloides* exhibited diauxic growth and glycerol consumption was inhibited by glucose^20^ while *Yarrowia lipolytica* simultaneously assimilated glucose and glycerol with higher assimilation rate for glycerol than glucose.^21,22^

*M. isabellina* strains are a type of attractive filamentous fungi for microbial oil production. They could accumulate high oil contents (60–80% oils of its dry cell mass), utilise a variety of carbon sources including single carbon sources (glucose, xylose, glycerol, fructose and acetate) as well as mixed carbon sources derived from lignocellulosic biomass, and tolerate relatively high levels of biomass degradation products such as furfural, HMF and phenolic compounds generated from pretreatment processes.^14,23–31^ Although *M. isabellina* strains have been extensively studied for microbial oil production in recent years, its ability to co-utilise glycerol–glucose mixture or glycerol–sugars derived from biomass for microbial oil production has not been studied.

Following microbial oil production, oils need to be recovered from oleaginous cell mass. In order to improve oil recovery from wet cell biomass, a number of methods have been used to pretreat oleaginous biomass prior to oil extraction, including enzymatic hydrolysis, chemical hydrolysis, bead milling, electroporation, high pressure homogenization and osmotic shock, microwave-induced heating, hydrothermal liquefaction (HTL) and employment of hydroxyl radicals generated from semiconductors (TiO_2_) under UV-light irradiation.^32^ Among these methods, HTL is a relatively simple, scalable and water-compatible thermal process conducted at a temperature range of 300–400 °C, which was also used for conversion of non-oleaginous biomass feedstocks such as lignocellulosic biomass and proteins/amino acids to “bio-oils” – a mixture consisting of phenols, esters, alcohols, aldehydes, ketones, *etc.*^33^ HTL has been extensively studied for the production and extraction of bio-oils including fatty acids from algae, which was well summarized by a recent publication.^34^ There are also a few HTL studies on yeast biomass,^34–39^ but the studies on HTL of filamentous fungal biomass for oil recovery is very limited.^40^

In this study, instead of using glycerol as a “drop-in” carbon source to improve microbial oil production from biomass pretreated by the glycerol-free methods, glycerol-based pretreatment was used to improve cellulose enzymatic digestibility, followed by microbial oil production on mixed substrates containing glucose and residual glycerol and HTL of fungal biomass for oil recovery. First, the effect of residual pretreatment hydrolysate (containing glycerol and biomass degradation products) from incomplete washed biomass on enzymatic hydrolysis by cellulases was investigated. Second, the ability of *M. isabellina* NRRL 1757 (ATCC 42613), one of the most studied *M. isabellina* strain for microbial oil production,^24–27,29^ on co-utilisation of biomass sugars and glycerol was assessed. Finally, a simple catalyst-free HTL process for direct pretreatment of fungal biomass in fermentation broth was evaluated for extraction and recovery of microbial oils. Integration of glycerol-based pretreatment, co-utilisation of pretreated biomass and residual glycerol and direct HTL of fungal biomass in fermentation broth may improve the overall process economics. The information achieved from this study is very useful for further development and optimization of this integrative microbial oil production process based on glycerol pretreatment of biomass.

## 2. Methods

### 2.1 Materials

Sugarcane bagasse (∼50% moisture) was collected from Racecourse Sugar Mill (Mackay, Australia) and was pretreated with acidified glycerol solution at QUT Mackay Renewable Biocommodities Pilot Plant located within the sugar mill. The detailed pretreatment procedure was described previously.^10^ Briefly, pretreatment was conducted at 150 °C for 15 min with the initial liquid/solid ratio of 6 : 1 (60 kg : 10 kg dry bagasse). The liquid contained ∼50 kg glycerol, ∼10 kg water and sulfuric acid (0.4% in liquid or 2.4% on dry bagasse). After pretreatment, solid and liquid were separated by press filtration, collected and stored separately at 4 °C. A portion of the solid residue was washed thoroughly to remove all the residual pretreatment hydrolysate, collected after vacuum filtration and stored at 4 °C for later use.

*M. isabellina* NRRL 1757 (Syn. *Umbelopsis isabellina* ATCC 42613) was obtained from the American Type Culture Collection (ATCC, Manassas, VA). *M. isabellina* NRRL 1757 was first cultured on potato dextrose agar to produce spores at 28 °C. After 5 days cultivation, the spores were washed from the agar using 0.9% NaCl–Tween solution as a spore suspension and maintained at 4 °C. Chemicals of analytical grade or above were purchased from Sigma-Aldrich (Australia).

### 2.2 Effect of pretreatment hydrolysate on enzymatic hydrolysis

Effect of glycerol-rich pretreatment hydrolysate on enzymatic hydrolysis of pretreated and washed bagasse was firstly conducted with addition of different amounts of pretreatment hydrolysate (corresponding to 0%, 2.0%, 5.0% and 7.5% (w/w) glycerol, respectively) to the enzymatic hydrolysis solution. Enzymatic hydrolysis was performed in 250 mL flasks containing 100 g reaction mixtures: 3% wt glucan (glycerol pretreated and washed bagasse), 0.5 mL Accellerase 1500/g glucan and required amount of pretreatment hydrolysate. The pH of the enzymatic solution was adjusted to 4.8 by the addition of 2 M NaOH. As control, pretreated and washed bagasse was also enzymatically hydrolyzed in citrate buffer solution (50 mM, pH 4.8) at the same conditions with addition of different amounts of pure glycerol (0, 2.0%, 5.0% and 7.5%, respectively). The experiments were conducted in duplicate at 140 rpm under 50 °C for 72 h. A small portion of samples (1 mL) were taken at 6 h, 24 h and 72 h for sugar analysis.

### 2.3 Preculture of *M. isabellina*

Preculture was conducted in a 250 mL shake flask containing 50 mL preculture medium consisting of 10.0 g L^−1^ glucose, 1.0 g L^−1^ (NH_4_)_2_SO_4_, 1.75 g L^−1^ KH_2_PO_4_, 0.5 g L^−1^ Na_2_HPO_4_, 1.5 g L^−1^ MgSO_4_·7H_2_O, 0.1 g L^−1^ CaCl_2_·2H_2_O, 0.008 g L^−1^ FeCl_3_·6H_2_O, 0.001 g L^−1^ ZnSO_4_·7H_2_O, 0.0001 g L^−1^ CuSO_4_·5H_2_O, 0.0001 g L^−1^ CoCl_2_·H_2_O, and 0.0001 g L^−1^ MnSO_4_·5H_2_O. Cultivation was started by inoculating 1 mL of spores solution containing 1 × 10^7^ spores to the shake flask and incubated at 28 °C with an agitation speed of 180 rpm for 48 h.

### 2.4 Effect of pretreatment hydrolysate on microbial oil production

Following enzymatic hydrolysis with addition of different amounts of pretreatment hydrolysate (0%, 2.0%, 5.0% and 7.5%, respectively), the hydrolysate was separated from solid by vacuum filtration and used as media with supplement of 1.0 g L^−1^ of KH_2_PO_4_ and 0.5 g L^−1^ of (NH_4_)_2_SO_4_. The pH of the media was adjusted to 6.0 with addition of 2 M NaOH before use. Microbial oil production was conducted at 28 °C and 180 rpm for 6 days in 250 mL flasks containing 45 mL enzymatic hydrolysates and 5 mL *Mortierella* preculture. At the end of the cultivation, fungal biomass and fermentation broth were separated by vacuum filtration. The fungal biomass was collected, washed, freeze-dried and stored at 4 °C. A portion of fermentation broth was stored at −20 °C for later analysis.

### 2.5 Kinetics of glucose and glycerol consumption by the *M. isabellina* strain

Pretreated and washed bagasse was firstly enzymatic hydrolyzed in 250 mL shake flasks containing 100 mL reaction mixture (3% glucan, 0.5 mL Accellerase 1500/g glucan, pH 4.8 (adjusted by 2 M NaOH)). Enzymatic hydrolysis was conducted at 50 °C and 140 rpm for 72 h. After enzymatic hydrolysis, liquid (enzymatic hydrolysate) and solid were separated by vacuum filtration. Pretreatment hydrolysate and pure glycerol were added to the enzymatic hydrolysate respectively to make cultivation medium containing 2.0% glycerol (∼20 g L^−1^). The cultivation medium was supplemented with 1.0 g L^−1^ of KH_2_PO_4_ and 1.0 g L^−1^ of (NH_4_)_2_SO_4_ while 0.7 g L^−1^ xylose was also added to the medium containing pure glycerol to mimic sugar composition in pretreatment hydrolysate. Microbial oil production was conducted at 28 °C and 180 rpm for 14 days in 250 mL flasks containing 45 mL enzymatic hydrolysates and 5 mL *Mortierella* preculture. During the cultivation, samples (whole flasks) were withdrawn every two days. Fungal biomass and fermentation broth were separated by vacuum filtration. The fungal biomass was collected, washed, freeze-dried and stored at 4 °C. A portion of fermentation broth was stored at −20 °C for later analysis.

### 2.6 Preparation of fungal biomass in a 1 L bioreactor

A 1.3 L stirred tank reactor (STR) with 1 L working volume was used to prepare sufficient *Mortierella* biomass and fermentation broth for HTL trials. Prior to cultivation, pretreated and washed bagasse was enzymatically hydrolyzed at 50 °C for 72 h in five 500 mL flasks containing 200 g reaction mixture: 7.5% glucan loading, pretreatment hydrolysate (5.0% glycerol), pH 4.8 and cellulose loading of 0.5 mL Accellerase 1500/g glucan. The resulting enzymatic hydrolysate was supplemented with 2.4 g L^−1^ of (NH_4_)_2_SO_4_ and 2.4 g L^−1^ of KH_2_PO_4_ to keep C/N and C/P ratios at similar levels to the shake flask trials in Section 2.4. In the other trial, 3.0% glucan loading and pretreatment hydrolysate (2%) were used for enzymatic hydrolysis, followed by addition of 1.0 g L^−1^ of KH_2_PO_4_ and 1.0 g L^−1^ of (NH_4_)_2_SO_4_ for microbial oil production. Microbial oil production was started with the inoculation of 100 mL *Mortierella* preculture (10%). The cultivation was conducted at 28 °C and the pH was maintained at 6.0 using 2 M NaOH and 1 M H_2_SO_4_. The DO level was maintained at above 20% by controlling the agitation speed (200–400 rpm) and the aeration rate (0.6–1.0 vvm). After 12 days of cultivation, fungal biomass and fermentation broth were separated by vacuum filtration, collected separately and stored at 4 °C for later use. A portion of fungal biomass was washed thoroughly to remove all the residual fermentation broth and was freeze-dried to determine the dry biomass content in the filtered fungal biomass.

### 2.7 Hydrothermal liquefaction (HTL) and bio-oil recovery

HTL trials were performed using a laboratory scale Hastalloy steel high pressure reactor (GC-3 gasket closure reactor) with a capacity of 285 mL. The reactor was fitted to a gas inlet/purge line with real time pressure monitor. Unwashed fungal biomass (containing 3.0 g dry biomass and 5.2 g fermentation broth) was firstly mixed with water or fermentation broth as solvent at a solid/liquid weight ratio of 1 : 30. The reactor was sealed and purged with argon three times to remove air and excess oxygen, followed by transferring the reactor to a fluidized sand bath preheated to 340 °C. The reactor was heated to 340 °C for 30 min, followed by HTL at 340 °C for 60 min. At the end of HTL, the reactor was cooled down by quenching it in an adjacent water bath. As control, 90 g of fermentation broth was liquefied at the same conditions without fungal biomass.

Once the reactor was cooled down to room temperature, the control valve was slowly opened to release the pressure. The reaction mixture was poured out and was adjusted to pH 2 with 5 M HCl solution to precipitate acid-insoluble components, followed by vacuum filtration using Whatman no. 1 filter paper. Diethyl ether (DEE) was used to wash the reactor and to extract bio-oils from the liquid and solid residues. The DEE extracts from the reactor, water and solid residue were mixed. The bio-oil weight was recorded after rotary evaporation of DEE solvent at 60 °C under vacuum and was used to calculate the yield based on initial dry weight of the fungal biomass.

### 2.8 Analytical methods

Microbial oil content in the dried fungal biomass was determined by the Bligh and Dyer method with modification.^41^ Briefly, about 40.0 mg of dried fungal biomass was weighed to a 2.0 mL centrifuge tube containing 4 stainless steel beads (1 bead with a diameter of 5.0 mm and 3 beads with a diameter of 2.5 mm). The tube was sealed with a screw-on lid and fungal biomass was lysed for 4 min by a tissue lyser (QIAGEN, US). Following lysis, 0.24 mL water, 0.3 mL chloroform and 0.6 mL methanol were added into the centrifuge tube, respectively. The mixture was further homogenised for 2.0 min, followed by centrifugation at 12 000 rpm for 2 min. After centrifugation, 0.3 mL chloroform was added to the centrifuge tube, followed by 0.5 min homogenisation and 5 min centrifugation at 12 000 rpm, respectively. The bottom layer was removed by a 1 mL syringe with a stainless steel needle and transferred to a pre-weighed HPLC vial. Another 0.3 mL chloroform was added to the centrifuge tube to repeat the extraction steps. The extraction was repeated 3 times (a total extraction times of 4) and the bottom layer solutions were transferred to the same HPLC vial. The solvent in the HPLC vial were evaporated out at 45 °C under vacuum and the oil weight was recorded to calculate the total content of microbial oil in fungal biomass.

For the microbial oil profile analysis, microbial oils were firstly converted to fatty acid methyl esters (FAMEs) by transesterification. Briefly, 100 mg of fungi biomass were reacted with methanol/hydrochloric acid/chloroform (10 : 1 : 1) in a glass tube at 90 °C for 60 min. After the glass tube was cooled down, 0.5 mL of 0.9% NaCl and hexane were added to the glass tube, respectively and mixed well. The hexane layer containing FAMEs was separated and transferred to a GC sample vial after centrifugation at 3900 rpm for 10 min using a bench-top centrifuge. Samples were analyzed using a Hewlett-Packard GC-MS system (HP6890 series GC with an HP5973 MS detector) with a HP-5MS capillary column (Agilent 30 m × 0.25 mm × 0.25 μm). They were injected with a split ratio of 90 : 1 into the injection port set at 260 °C. The temperature program commenced at 90 °C, raised at 4 °C min^−1^ until 200 °C, then raised at 1 °C min^−1^ until 260 °C and hold for 5 min. The bio-oil samples from HTL trials were analyzed using the same GC-MS system. DEE fraction samples were injected with a split ratio of 50 : 1 into the injection port set at 290 °C. The column temperature was initially maintained at 90 °C for 10 min before increasing to 290 °C at a heating rate of 5 °C min^−1^. The carrier gas for GC-MS analysis was helium at a flow rate of 1 mL min^−1^.

Sugars, organic acids and sugar degradation products in pretreatment hydrolysate, enzymatic hydrolysate and fermentation broth were quantified by HPLC methods as described previously.^10^ Total phenolics in the enzymatic hydrolysate medium was determined using the Folin–Ciocalteau micro method adapted by Andrew Waterhouse.^31,42^ The total phenolic contents were calculated on the basis of the calibration curve of gallic acid and expressed as gallic acid equivalents (GAE), in milligrams per milliliter of the sample.

## 3. Results and discussion

### 3.1 Effect of pretreatment hydrolysate on enzymatic hydrolysis

**Fig. 1:**
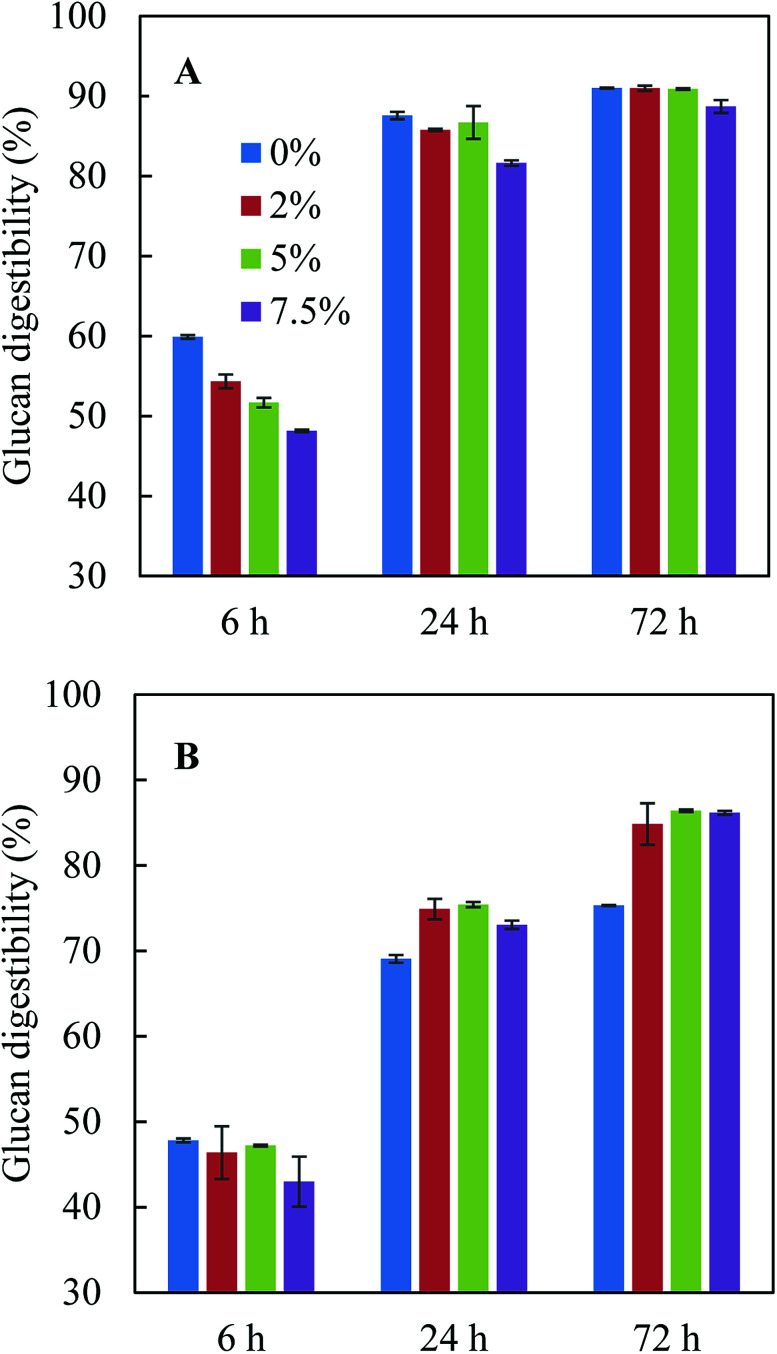
Effect of glycerol-rich pretreatment hydrolysate (0%, 2%, 5% and 7.5% glycerol) on enzymatic hydrolysis. (A) In citric buffer; (B) with pretreatment hydrolysate.

The pretreatment hydrolysate obtained after acid-catalysed glycerol pretreatment of sugarcane bagasse consisted of 444.8 g kg^−1^ glycerol, 3.5 g kg^−1^ glucose, 12.0 g kg^−1^ xylose, 4.8 g kg^−1^ acetate, 2.9 g kg^−1^ furfural and 0.3 g kg^−1^ HMF. The dilution of glycerol (∼830 g kg^−1^ at the beginning of pretreatment) was due to the addition of condensate during pretreatment. Fig. 1 shows the effect of pure glycerol in citrate buffer and residual glycerol in pretreatment hydrolysate on enzymatic hydrolysis. When citrate buffer was used (Fig. 1A), addition of pure glycerol inhibited glucan digestibility in the first 6 h and the inhibition became more significant when the glycerol concentration increased from 0% to 7.5%. However, after 24 h and 72 h hydrolysis, the glucan digestibilities were similar with adding 0–5% pure glycerol though the glucan digestibilities were slightly lower with 7.5% pure glycerol. The observation from this study was different from the previous study,^10^ in which it was found that the presence of 5.0% pure glycerol inhibited glucan digestibility. The different observations were possibly due to the difference in glucan loading: 3% glucan loading was used in this study while 5% loading was used in the previous study.^10^ Indeed, the previous study found that although 15% glycerol could reduce glucan digestibility by 19.5% at an initial glucan loading of 7.5%, dilution of the glycerol concentration in the enzymatic hydrolysis solution to 5% glycerol (corresponding to a glucan loading of 2.5%) with citrate buffer increased glucan digestibility to the level close to that in citrate buffer but without glycerol.^10^ These results indicated both glycerol concentration and glucan loading could affect enzymatic hydrolysis.

When glycerol-rich pretreatment hydrolysate was added to the enzymatic hydrolysis solution without citrate buffer, the 72 h glucan digestibilities at the same glycerol concentrations (2.0–7.5%) were 3–5% lower than those with pure glycerol with citrate buffer (Fig. 1A and B), which was possible as the addition of citrate buffer can help to maintain optimal pH range for enzyme hydrolysis and to achieve the best glucan digestibility. Although a high glycerol concentration of 7.5% seemed to inhibit enzymatic hydrolysis in the first 24 h, the glucan digestibilities at 72 h with all glycerol concentrations were similar (Fig. 1B). When citrate buffer was not present, adding pretreatment hydrolysate (up to 7.5% glycerol) led to 10–11% higher glucan digestibility after 72 h hydrolysis compared to control without pretreatment hydrolysate (Fig. 1B). It was also noted that with the addition of pretreatment hydrolysate, the pH of the reaction solution only changed from 4.8 to 4.3 after 72 h hydrolysis, while the pH dropped to 3.9 in control without addition of pretreatment hydrolysate or citrate buffer. The lowest glucan digestibility observed in control in fact indicated that pretreatment hydrolysate may have some buffering effect due to the presence of organic acid salts such as acetate^43^ and the buffering effect may partially offset the inhibition effect from glycerol, resulting in higher glucan digestibilities in the presence of pretreatment hydrolysate (Fig. 1B).

### 3.2 Effect of pretreatment hydrolysate on microbial oil production by *M. isabellina*

Furthermore, the effect of glycerol-rich pretreatment hydrolysate on microbial oil production was investigated. The pretreatment hydrolysate had a very high concentration of glycerol (444.8 g kg^−1^) and a higher-than-water density (∼1.11 g mL^−1^). In addition, the pretreatment hydrolysate also contained soluble sugars (3.5 g kg^−1^ glucose and 12.0 g kg^−1^ xylose). As a result, addition of pretreatment hydrolysate to the enzymatic hydrolysate to prepare media with glycerol concentrations of 0–7.5% did not dilute sugars concentrations much as shown in Table 1. The initial concentrations of glucose and xylose were ∼25.9–27.0 g L^−1^ and 1.5–2.7 g L^−1^, respectively.

**Table 1:** Concentrations of monosaccharide in the media containing 0–7.5% glycerol (g L^−1^)

Monosaccharide	Medium composition (day 0 after inoculation)			
	0%	2%	5%	7.5%
Glucose	25.9	27.0	26.3	26.7
Xylose	1.4	1.5	2.0	2.4
Glycerol	0.0	18.6	44.5	68.5
Acetic acid	0.1	0.5	1.1	1.8
Furfural	0.0	0.1	0.3	0.5
HMF	0.0	0.01	0.03	0.05
Total phenolics	0.0	0.3	0.8	1.1
C/N	56 : 1	98 : 1	141 : 1	178 : 1

After 6 days cultivation, the amounts of consumed carbon sources, biomass concentrations, microbial oil production under different amounts of pretreatment hydrolysate (corresponding to different concentrations of glycerol) are shown in Table 2. Small portions of glycerol (3.4–4.2 g L^−1^) were consumed with initial glycerol concentrations of 2–7.5%. Increasing glycerol concentration (and compounds derived from biomass as shown in Table 1) inhibited glucose (and xylose) consumption though the overall fungal biomass was not affected up to 5% glycerol. However, biomass concentration reduced when glycerol concentration further increased to 7.5%. The reduced fungal biomass concentration was possibly due to the inhibition on the growth of the strain by glycerol and/or biomass degradation compounds. Several studies have showed the inhibitions of biomass degradation products at elevated levels on the growth of oleaginous microorganisms including yeast and filamentous fungi.^26,44,45^ Especially, a previous study with the same *Mortierella* strain showed that although the strain could tolerate up to relatively high levels of degradation compounds (up to 2 g L^−1^ furfural, 0.4 g L^−1^ HMF and some trace phenolic compounds) derived from biomass, the growth rate was reduced by 28%.^26^ Although the furan (and possibly soluble phenolics) concentrations in this study were much lower than those in that previous study, there may be a synergetic inhibition on fungal growth from glycerol and biomass degradation compounds.

**Table 2:** Effect of pretreatment hydrolysate on microbial oil production using the *M. isabellina* strain grown on pretreated bagasse enzyme hydrolysate^a^

Samples	Consumed carbon source (g L^−1^)				Fungal biomass (g L^−1^)	Microbial oils		
	Glucose	Xylose	Glycerol	Acetate		Content (%)	Concentration (g L^−1^)	Yield (g g^−1^ consumed carbon sources)
EH	19.2 ± 0.7	0.8 ± 0.0	—	0.1 ± 0.0	7.7 ± 0.2	49.6 ± 0.3	3.8 ± 0.1	0.19 ± 0.00
EH + 2% PH	16.7 ± 0.4	0.5 ± 0.1	4.2 ± 0.3	0.5 ± 0.0	7.2 ± 0.4	52.3 ± 0.1	3.8 ± 0.2	0.14 ± 0.01
EH + 5% PH	14.8 ± 0.2	0.3 ± 0.0	3.4 ± 0.0	1.1 ± 0.0	7.8 ± 0.1	50.3 ± 0.5	3.9 ± 0.1	0.16 ± 0.01
EH + 7.5% PH	12.6 ± 0.2	0.4 ± 0.0	4.2 ± 1.6	1.8 ± 0.0	6.7 ± 0.1	47.0 ± 0.6	3.1 ± 0.0	0.12 ± 0.01

a Experiments were conducted at 28 °C and 180 rpm for 6 days. Data points are the average of two replicates with standard deviations.

It was also observed that increasing glycerol concentration from 0% to 2.0% led to a slight increase in oil content from 49.6% to 52.3% (Table 2). The increase in oil content was possibly due to the increase in C/N ratio from 56 : 1 to 98 : 1 (Table 1) as it is well known that higher C/N ratio promotes the accumulation of microbial oils.^31,46^ However, further increasing glycerol content to 7.5% resulted in oil content decrease to 47.0% though the C/N ratio further increased from 98 : 1 to 118 : 1. The drop in oil content was possibly caused by the increased levels of biomass degradation products from pretreatment hydrolysate (Table 1). Previous studies showed that high furan concentrations (especially furfural) and phenolic compounds could reduce the oil content in yeast^47^ and *M. isabellina* biomass (though the decrease was not statistically significant).^26^ In this study, with 7.5% glycerol, the concentrations of furfural, HMF and total phenolics reached to 0.5 g L^−1^, 0.1 g L^−1^ and 1.1 g L^−1^ respectively, which may have caused synergetic inhibition from biomass degradation products (and possibly glycerol) on the synthesis of microbial oils.

A recent study showed that carbon source was channeled to both cellular microbial oils and polysaccharide synthesis in the early oleaginous phase in the growth of *Mortierella isabellina*, followed by conversion of both glucose and polysaccharides to microbial oils during transition from the early to the late oleaginous phase.^48^ Although the mechanisms of the effect of inhibitors on carbon flux distribution has not been well understood, the reduced oil content possibly is accompanied with the increase in cellular polysaccharides.

Microbial oil concentration was determined by fungal biomass concentration and microbial oil content. As shown in Table 2, microbial oil concentration was not affected by glycerol concentration up to 5% and only reduced at a glycerol concentration of 7.5% due to the inhibition of fungal growth and reduction in oil content as discussed above. Regarding the oil yield on consumed carbon sources, there was no clear trend on the change. Nevertheless, it is generally accepted that the theoretical oil yield on glycerol is higher than that on glucose (∼0.34 *vs.* ∼0.32 g g^−1^).^49^ With the use of mixed glucose–glycerol carbon sources, intermediate oil yields were achieved by a yeast strain in a previous study.^20^

Table 3 shows fatty acid profiles with different concentrations of glycerol from pretreatment hydrolysate. The oils were dominated by oleic acid (57–60%, C18:1), palmitic acid (17–19%, C16:0) and linoleic acid (11–12%, C18:2) (Table 3). The overall composition was in line with previous reports with *M. isabellina* strains.^14,24,26,28–30,47^ It appeared that the contents of C18:1 decreased slightly while C18:0 increased slightly with increasing glycerol concentration to 7.5%. In previous studies, it was found that the carbon source types and biomass degradation products affected fatty acid profiles of yeast and filamentous fungi including *M isabellina*.^47^ For example, the use of molasses as a carbon source by *M. isabellina* ATHUM 2935 led to a higher percentage of C18:2 than those achieved with fructose and glucose while the percentage of C16:0 had a reverse trend.^28^ A higher percentage of C18:0 was observed with glucose than that with xylose with *M. isabellina* ATHUM 2935.^30^ With *M. isabellina* NRRL 1757, a lower percentage of C18:0 but a higher percentage of C18:1 were observed with glucose than xylose.^24^ Furthermore, a lower percentage of C18:1 but higher percentages of C16:0, C18:0 and C18:2 were reported with the ATHUM 2935 strain.^14^ In addition, Ruan *et al.*^26^ reported decreased percentages of C18:1, C18:3 and C16:1 but increased percentages of C18:0 and C16:0 with the *M. isabellina* NRRL 1757 strain in the presence of biomass degradation products such as furans and soluble phenolics, indicating that the inhibitors may cause the desaturation of the fatty acid desaturation. Zeng *et al.*^24^ reported increased percentages of C18:1 but decreased C16:1 in the presence of vanillin and furfural with *M. isabellina* NRRL 1757. Different observations on fatty acid profiles in this study possibly were the results generated from synergetic effects from carbon sources and biomass degradation products. It is worth noting that the *M. isabellina* strain also produced γ-linolenic acid (C18:3), a medically important high value fatty acid. The γ-linolenic acid percentages in this study at different glycerol concentrations were in line with the reported range of 1–7% with *M. isabellina* strains.^14,24,26,28–30^

**Table 3:** Fatty acid composition of the *M. isabellina* strain grown on pretreated bagasse enzyme hydrolysate with or without the addition of pretreatment hydrolysate

Samples	Relative fatty acid content (%)						
	C16:0	C16:1	C18:0	C18:1	C18:2	C18:3	C20:0
EH	18.4 ± 0.0	2.3 ± 0.0	3.6 ± 0.0	60.0 ± 0.4	11.3 ± 0.1	3.0 ± 0.1	1.2 ± 0.0
EH + 2% PH	17.0 ± 0.2	1.8 ± 0.0	4.7 ± 0.2	59.7 ± 0.2	12.5 ± 0.1	2.9 ± 0.0	1.4 ± 0.0
EH + 5% PH	17.6 ± 0.2	1.7 ± 0.0	4.8 ± 0.1	59.2 ± 0.9	11.4 ± 0.0	3.2 ± 0.0	1.6 ± 0.1
EH + 7.5% PH	19.0 ± 0.4	1.7 ± 0.0	5.5 ± 0.0	57.4 ± 0.9	11.1 ± 0.2	3.2 ± 0.1	1.6 ± 0.0

### 3.3 Kinetics of glucose and glycerol consumption by the *M. isabellina* strain

From the above results, it appeared that enzymatic hydrolysate with 2% glycerol could achieve a better yield of biomass and microbial oils. Thus pretreatment hydrolysate was added to the enzymatic hydrolysate to make a final glycerol concentration of 2% and used for preparing media for microbial oil production, as well as investigating how glycerol can be assimilated at the present of glucose by the *M. isabellina* strain. As a control, pure glycerol at equivalent concentration was added instead of pretreatment hydrolysate. Table 4 shows the composition of the media used in this study. Both media had similar levels of sugars and phosphorous and nitrogen sources. For medium containing pure glycerol, xylose was also supplemented to simulate the composition of sugars in the medium containing pretreatment hydrolysate.

**Table 4:** Composition of the media containing pretreatment hydrolysate and pure glycerol (g L^−1^)

Composition	Medium containing pretreatment hydrolysate (PH) (day 0)	Medium containing pure glycerol (PG) (day 0)
Glucose	27.6	26.8
Xylose	1.3	1.6
Glycerol	21.2	19.7
(NH_4_)_2_SO_4_	0.9	0.9
KH_2_PO_4_	0.9	0.9

**Fig. 2:**
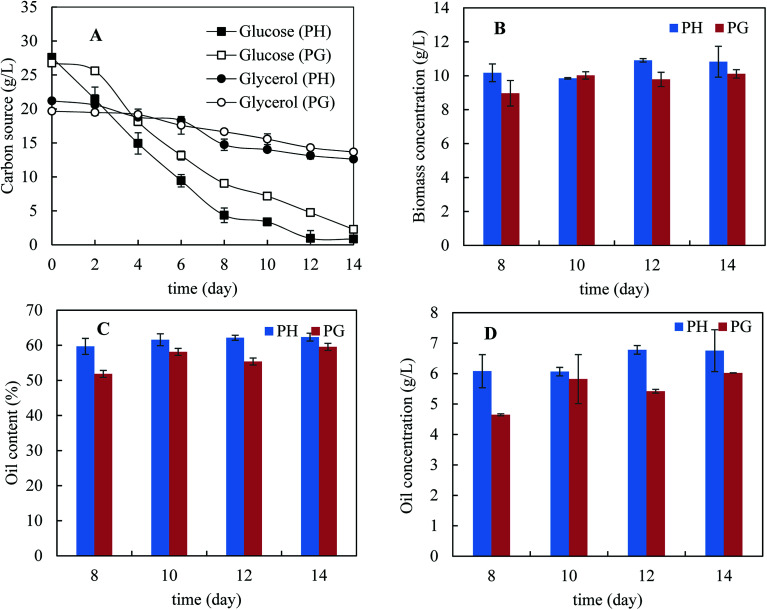
Fermentation profile of the *M. isabellina* strain grown on pretreated bagasse enzyme hydrolysate with pretreatment hydrolysate and pure glycerol, respectively. (A) Kinetics of carbon source consumption; (B) fungal biomass concentrations; (C) oil contents; (D) oil concentrations. PH: pretreatment hydrolysate; PG: pure glycerol.

Fig. 2A shows the kinetics of consumption of major carbon sources – glucose and glycerol. Xylose consumption was not present because of its low concentrations (less than 2 g L^−1^). It was noticed that glucose consumption was much faster than glycerol consumption, indicating catabolic repression of glycerol by the present of glucose. Interestingly, glucose was consumed more rapidly in the presence of pretreatment hydrolysate than that in pure glycerol and the same was observed for glycerol consumption. This indicates that biomass degradation products did not inhibit glucose consumption. In addition, glycerol consumption with pretreatment hydrolysate was also slightly faster than that with pure glycerol. The higher consumption rate of carbon sources in the presence of pretreatment hydrolysate was possibly because of more diversified micronutrients presented in pretreatment hydrolysate. Fig. 2B–D shows the biomass concentration, microbial oil content and microbial oil concentration from day 8 to 14. More biomass and higher microbial oil content were achieved with the pretreatment hydrolysate-containing medium than that with pure glycerol containing medium. As a result, higher microbial oil concentrations were achieved in the presence of pretreatment hydrolysate. After 14 days cultivation, only 8.6 and 6.0 g L^−1^ glycerol were used in the media supplemented with pretreatment hydrolysate and pure glycerol, respectively, corresponding 40% and 30% of the initial glycerol concentrations, respectively. In order to improve glycerol consumption, addition of extra ammonium sulphate up to 2 g L^−1^ was studied but the improvement in glycerol consumption was limited despite extended fermentation time (data not shown).

Co-utilisation of sugars–glycerol mixture by *M. isabellina* was not reported so far though as a sole carbon source, it was studied for microbial oil production by *M. isabellina* strains.^14,30,46^ There are several recent studies on co-utilisation of sugars–glycerol mixtures for production of microbial oils and other compounds by yeast strains,^20,21,50,51^ which may help to understand the glycerol metabolic mechanisms in the presence of glucose. With *Yarrowia lipolytica* IBT 446, it was reported that glycerol was utilised faster than glucose though both carbon sources were consumed simultaneously.^21^ With *Rhodosporidium toruloides* DSMZ 4444, diauxic growth on glycerol and glucose was reported and consumption of glycerol started after depletion of glucose.^20,50^ Glycerol kinase is an important enzyme in glycerol assimilation and catabolizes glycerol to glycerol-3-phosphate, a precursor for fatty acid biosynthesis.^21,50^ With *Saccharomyces cerevisiae*, the presence of glucose completely repressed the transcription of the genes encoding glycerol kinase.^51^ With *Rhodosporidium toruloides* DSMZ 4444, however, the presence of glucose did not cause the down-regulation of glycerol kinase despite the inhibition of glycerol assimilation.^20,50^ The glycerol metabolic mechanism still has not been well understood with the *Rhodosporidium toruloides* yeast. With *M. alpina*, it was reported that the presence of glucose repressed the expression of glycerol kinase glucose after nitrogen exhaustion but it was still unclear how the strain metabolizes a dual carbon source of glucose–glycerol.^52^ The kinetics of carbon source consumption (glucose preferred but simultaneous consumption of both) by *M. isabellina* indicate a different mechanism of co-utilisation of glycerol–glucose from previous mechanisms (glycerol preferred but simultaneous consumption of both and glucose preferred with diauxic growth pattern) with yeast strains.

Considering the challenges (the high glycerol concentration and the formation of xylosides)^8^ in recovery of xylose as carbon source and slow consumption of glycerol based on the one-step glycerol pretreatment process, a two-step microbial oil production process based on a two-step pretreatment (1^st^ dilute acid – 2^nd^ acidified glycerol pretreatment) strategy is being investigated to improve the xylose recovery and utilization efficiency of both xylose and glycerol in another study.

### 3.4 Preparation of fungal biomass in a 1 L bioreactor

**Fig. 3:**
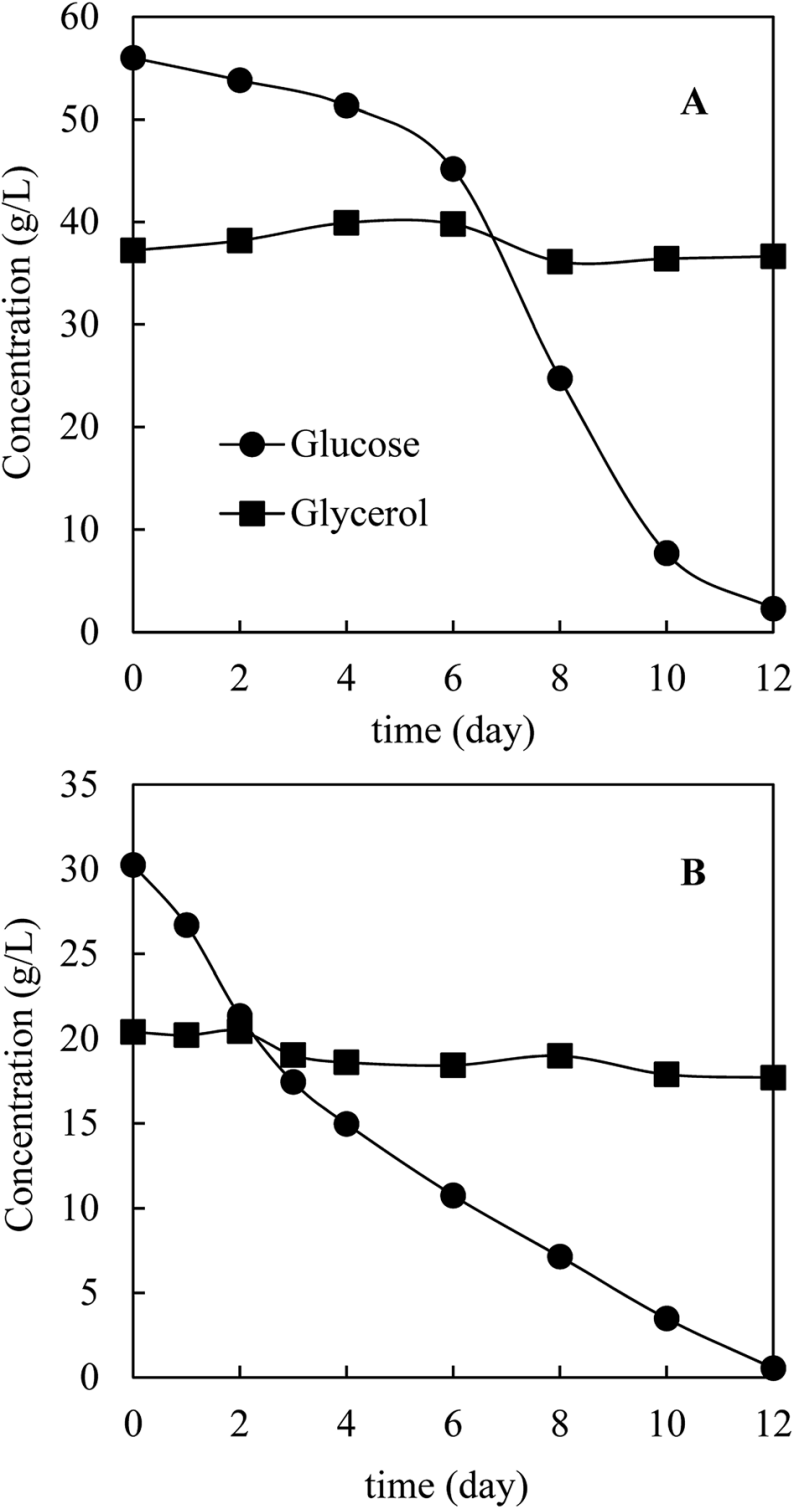
Kinetics of carbon source consumption in a 1 L bioreactor. (A) Initial carbon sources: 56.0 g L^−1^ glucose + 37.2 g L^−1^ glycerol; (B) 30.2 g L^−1^ glucose and 20.4 g L^−1^ glycerol.

Prior to HTL, *M. isabellina* was cultivated in a 1 L bioreactor with sugars and glycerol derived from bagasse pretreatment to achieve sufficient fungal biomass. The medium (day 0) contained 56.0 g L^−1^ glucose, 37.2 g L^−1^ glycerol, 2.6 g L^−1^ xylose and 0.9 g L^−1^ acetic acid, respectively. Fig. 3 shows the kinetics of carbon source consumption. After 12 days cultivation, almost all the glucose (53.7 g L^−1^) was consumed while surprisingly only 0.7 g L^−1^ glycerol was consumed. In addition, approximately 2.9 g L^−1^ of trace carbon sources (acetic acid and xylose) were consumed. As a result, 19.6 g L^−1^ biomass was harvested with an oil content of 64.0 ± 4.7%, an oil concentration of 12.5 ± 0.9 g L^−1^ and an oil yield of 0.22 ± 0.02 g g^−1^ consumed carbon sources. The oil concentration was comparable with the reported one (12.7 g L^−1^) from ∼68 g L^−1^ commercial glucose after 160 h cultivation in a 3 L bioreactor by *M. isabellina* ATHUM 2935 ^28^ and was much higher than that (6.9 g L^−1^) achieved after 184 h cultivation in a 7.5 L bioreactor using sugars derived from corn stover hydrolysates (28.6 g L^−1^ glucose and 16.1 g L^−1^ xylose) by the same *M. isabellina* NRRL 1757 strain (ATCC 42613).^25^ The oil yield on consumed carbon sources was relatively high but in line with the reported (or calculated) yields with the *M. isabellina* strains.^30,53^ In order to find out the reason why glycerol was barely consumed, the other reactor cultivation was conducted with the sugars and glycerol concentrations (∼3% glucose and ∼2% glycerol) same as those for the shake flask cultivation. As shown in Fig. 3B, after 12 days cultivation, almost all the glucose was consumed with the production of 10.8 g L^−1^ fungal biomass. However, only 2.7 g L^−1^ glycerol was consumed compared to 8.1 g L^−1^ glycerol consumption in shake flask after 12 days cultivation (Fig. 2). The much lower glycerol consumption in reactor cultivations was possibly related to better oxygen supply in reactors, leading to more preferable use of glucose for fungal growth and lipid production and further inhibition of glycerol consumption. Interestingly, in the cultivation of *Yarrowia lipolytica*, a strain preferably uses glycerol than glucose, better oxygen supply improved glycerol consumption and further inhibited glucose assimilation.^21,22^ These different observations further indicate different metabolic mechanisms of microorganisms on glycerol–glucose mixtures. In addition, the *M. isabellina* strain might also produce glycerol from glucose, which was observed with a oleaginous *Rhodosporidium* yeast,^20^ which may also explain the slight increase in glycerol concentration for the first 6 days in Fig. 3B.

### 3.5 HTL of fungal biomass to recover microbial oils

**Fig. 4:**
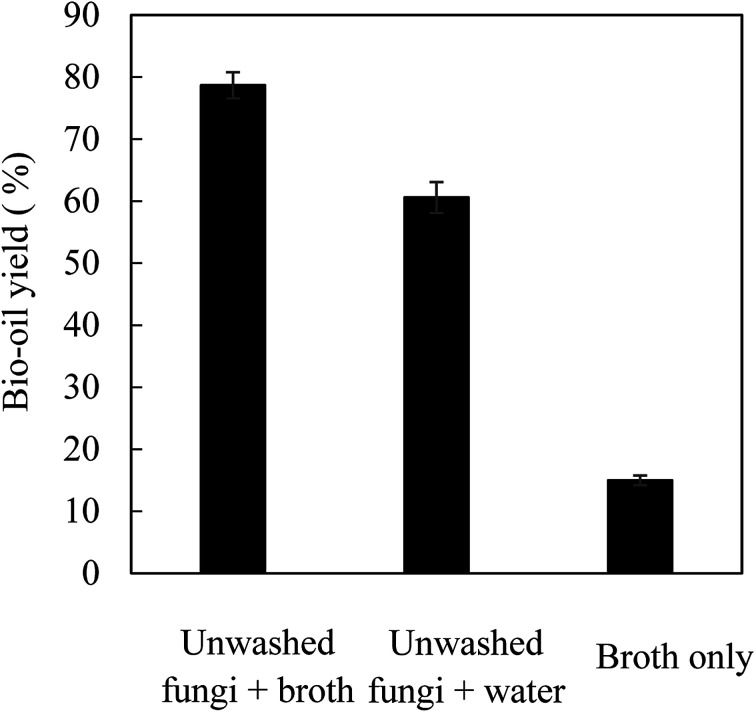
Yield of bio-oils after HTL and DEE extraction.

HTL was conducted with the use of the fungal biomass harvested from the first reactor cultivation. Fig. 4 shows the yield of bio-oils following HTL and processing. The highest oil yield of 78.7% (based on dry fungal biomass) was achieved from HTL of unwashed fungal biomass in fermentation broth while the oil yield from HTL of unwashed fungal biomass (containing 5.2 g fermentation broth) in water was 60.6%. The higher bio-oil yield obtained with unwashed fungal biomass in fermentation broth was mainly attributed to the conversion of some organic matters in fermentation broth to bio-oils. As shown in Fig. 4, HTL of fermentation broth alone contributed to 15.0% yields of bio-oils when compared to fungal biomass. This explained the high oil yield when HTL was conducted in fermentation broth compared to that in water. Based on the classic Bligh & Dyer extraction method, the washed fungal biomass contained 64.0% microbial oils and HTL of fungal biomass achieved a “true” bio-oil yield of 63.7% (78.7% minus 15.0%) (Fig. 4).

HTL has been extensively studied as a pretreatment method to assist microbial oil extraction from algae and for direct conversion of non-fatty acid components (protein, lignin, carbohydrates, *etc.*) of biomass to bio-oils, which have been well summarized in recent publications.^32–34^ There are also a few studies on HTL of microbial biomass for the production of bio-oils with the focus on yeast biomass.^34–40^ Although HTL does not require catalysts, alkaline catalysts such as Na_2_CO_3_, K_2_CO_3_ and KOH are widely used to improve the oil yields because they promote the formation of aromatic oils.^34^ In general, the oil yields obtained from HTL of algae and microbial cell biomass are less than 50% because of the low fatty acid contents (generally less than 30% in cell mass) and the inefficient conversion of non-fatty acid components (mainly carbohydrates and proteins) to oils.^34,40^ For example, HTL of microalgae *Dunaliella tertiolecta* biomass containing 39–61% proteins and 2.9–5.4% microbial oils (the content variations caused by the measurement method) at 360 °C for 50 min only achieved a maximum bio-oil yield of 25.8% in the presence of 5% Na_2_CO_3_.^54^ In another study, HTL of *Saccharomyces* yeast containing 75% proteins and 17% carbohydrates at 350 °C for 60 min only produced ∼32% bio-oils in the absence of catalyst.^36^ Recently, HTL of *Rhizopus* cell mass containing 34.2% proteins, 34.8% carbohydrates and 22.4% microbial oils at 300 °C for 19 min led to a bio-oil yield of 60% without catalyst.^40^ So far, the reported highest bio-oil yield from HTL of algae and microbial cell mass is possibly from the HTL of defatted *Cryptococcus* yeast biomass consisting of 44.9% proteins, 26.0% carbohydrates and 24.0% microbial oils, which led to a bio-oil yield of 68.9% at 350 °C with 1 M K_2_CO_3_ and 67.0% at 300 °C with 1 M KOH, respectively.^34^

The profiles of major fatty acids of microbial oils and bio-oils obtained from HTL of fungal biomass were compared and shown in Table 5. The *Mortierella* strain used in this study contained 64% microbial oils (fatty acids), only 9.0% crude proteins (data not shown) and others (not determined). HTL of unwashed fungi (containing a negligible amount of broth) in water led to an oil yield of 60.6% (Fig. 4) with fatty acids (C16:0, C18:1 and C18:0) are the predominant components (Table 5). This indicates that the non-fatty acid components in the cell biomass were barely converted to bio-oils. Based on the percentage (93.4%) of major fatty acids in the bio-oils, it was estimated the microbial oil (fatty acids) recovery was 91% compared to the majority of microbial oil components (C16:0, C18:0, C18:1, C18:2 and C18:3) in the fungal biomass. For the HTL in fermentation broth, the three fatty acids (C16:0, C18:1 and C18:0) accounted for 88.8% of the total bio-oils and the total microbial oil recovery was 113% based on the oil yield of 78.7% in comparison of initial amount of microbial oils in the fungal biomass (based on FAMEs analysis). The higher-than-100% microbial oil recovery was possibly due to the contribution of microbial oils from fermentation broth as shown in Table 5 (C16:0), under-estimation of microbial oils using FAMEs analysis. In addition, the fermentation broth contained glycerol (∼4%) and metabolites, which may also lead to more efficient cell mass deconstruction and microbial oil recovery. Although other non-microbial oil components in fungal cell mass were barely converted to bio-oils, the oils dominated by fatty acids may be more suitable for the production of high quality and stable biofuels than the bio-oils with diversified oil profiles and impurities such as nitrogen (mainly derived from proteins).

**Table 5:** Profiles of major oil components

Component	Without HTL (based on FAMEs)	HTL (unwashed + water)	HTL (unwashed + broth)	HTL (broth)
Phenol			1.2	14.3
2,3-Dimethyl-2-cyclopenten-1-one			1.3	19.9
Phenol, 3-methyl-				14.9
Phenol, 2-methoxy-				12.9
Phenol, 4-ethyl-				7.5
4-Isopropylthiophenol				7.2
Silicic acid				18.7
*n*-Hexadecanoic acid (palmitic acid, C16:0)	20.3	17.6	17.0	3.4
Octadecanoic acid (stearic acid, C18:0)	8.5	12.8	14.0	
9-Octadecenoic acid (oleic acid, C18:1)	49.1	63.0	57.8	
9,12-Octadecadienoic acid (linoleic acid, C18:2)	14.5			
6,9,12-Octadecatrienoic acid	4.7			
(γ-Linolenic acid, C18:3)				
Total	97.1	93.4	91.3	98.7

Compared to the fatty acid profiles of fungal biomass from shake flasks (Table 3), the percentage of C18:1 significantly reduced from ∼57–59% (day 6 in shake flasks) to 49% (day 12 in the reactor) while the percentages of C18:2 and C18:3 fatty acids increased. The fatty acid profile change was possibly related to the changed cultivation conditions (longer cultivation time, reactor cultivation, changed oxygen supply, *etc.*) but needs to be confirmed in future. The concentration of C18:3, namely, γ-linolenic acid was 588 mg L^−1^ out of 12.5 g L^−1^ total oils, comparable to 596 mg L^−1^ out of 17.8 g L^−1^ oils in shake flasks and higher than 508 mg L^−1^ of 12.7 g L^−1^ oils achieved in a bioreactor using pure glucose by *M. isabellina* ATHUM 2935.^28,30^

In addition, it was also noted that after HTL of fungal biomass, less saturated fatty acids became more saturated ones. As shown in Table 5, enrichment of C18:0 (stearic acid) and C18:1 (oleic acid) was accompanied with the disappearance of C18:1 (linoleic acid) and C18:2 (γ-linolenic acid). The loss of unsaturated fatty acids containing two and three double bonds after HTL was possibly due to the hydrogenation of these fatty acids during HTL.^55^ Under the HTL condition, water, glycerol and organic acids remaining in the fermentation broth could act as hydrogen donors^38^ and hydrogenation may have occurred *in situ* with saturation of the most unsaturated fatty acids to less unsaturated fatty acids.^55^

The bio-oils from HTL of fermentation broth were dominated with phenols, which presumably derived from bagasse lignin. The phenols accounted for 57% of the total oils, followed by a 2,3-dimethyl-2-cyclopenten-1-one (19.9%) – a component possibly derived from the bagasse carbohydrates, and then silicic acid (18.7%). Only phenol and 2,3-dimethyl-2-cyclopenten-1-one was detected in the bio-oils from HTL of fungal biomass in fermentation broth, possibly indicating the transformation to others and/or absorbance of these components on the solid residue after HTL. Absence of silicic acid in oils will be good for the quality of fuels (biodiesel or jet fuels) as silicic acid can be a source of ash, which could be emitted form engine as harmful particulate matter.

## 4. Conclusion

This study demonstrated an integrative process for microbial oil production consisting of pretreatment of sugarcane bagasse by acidified glycerol, enzymatic hydrolysis of pretreated bagasse in the presence of pretreatment hydrolysate containing residual glycerol, co-utilisation of biomass sugars and glycerol for microbial oil production by *M. isabellina* NRRL 1757 and HTL of fungal biomass in fermentation broth in the absence of catalyst for microbial oil recovery. The residual glycerol-rich pretreatment hydrolysate had little effect on enzymatic hydrolysis with glycerol concentrations up to 7.5%. The residual pretreatment hydrolysate with glycerol concentrations up to 5.0% did not affect microbial oil production. Reactor cultivation of *M. isabellina* NRRL 1757 increased glucose consumption but reduced glycerol assimilation. The mechanisms behind co-utilisation of glucose and glycerol by *M. isabellina* NRRL 1757 need to be studied, which may lead to the development of more efficient co-utilisation strategies for microbial oil production by this strain. A simple HTL process without a catalyst could efficiently recover microbial oils from fungal biomass in fermentation broth with the enrichment of more saturated fatty acids.

## Conflicts of interest

There are no conflicts to declare.

## Supplementary Material
